# Adding Natural Areas to Social Indicators of Intra-Urban Health Inequalities among Children: A Case Study from Berlin, Germany

**DOI:** 10.3390/ijerph13080783

**Published:** 2016-08-04

**Authors:** Nadja Kabisch, Dagmar Haase, Matilda Annerstedt van den Bosch

**Affiliations:** 1 Department of Geography, Humboldt-Universität zu Berlin, Berlin 10099, Germany; nadja.kabisch@geo.hu-berlin.de (N.K); dagmar.haase@geo.hu-berlin.de (D.H.); 2 Department of Ecosystem Services, Helmholtz Centre for Environmental Research—UFZ, Leipzig 04318, Germany; 3 German Centre for Integrative Biodiversity Research (iDiv) Halle-Jena-Leipzig, Leipzig 04103, Germany; 4 Department of Computational Landscape Ecology, Helmholtz Centre for Environmental Research—UFZ, Leipzig 04318, Germany; 5 Department of Work Science, Business Economics and Environmental Psychology, Swedish University of Agricultural Sciences, Alnarp 23053, Sweden; matilda.van.den.bosch@slu.se; 6 School of Population and Public Health and Department of Forest and Conservation Sciences, University of British Columbia, Vancouver, BC V6T 1Z3, Canada

**Keywords:** health inequality, children’s health, green space, intra-urban, overweight, measles, cluster analysis

## Abstract

Research suggests that there is a relationship between the health of urban populations and the availability of green and water spaces in their daily environment. In this paper, we analyze the potential intra-urban relationships between children’s health determinants and outcomes and natural areas in Berlin, Germany. In particular, health indicators such as deficits in viso-motoric development in children are related to environmental indicators such as the natural area cover, natural area per capita and distance to natural areas; however, these indicators are also correlated with social determinants of health. The methodological approach used in this study included bivariate and multivariate analyses to explore the relations between health inequalities and social, socio-economic, and land use parameters. The results on a sub-district level indicated that there was a correlation between natural areas and social health determinants, both of which displayed a certain intra-urban spatial pattern. In particular, a lower percentage of natural area cover was correlated with deficits in viso-motoric development. However, results with percentage of natural area cover and per capita natural area with childhood overweight were not conclusive. No significant correlation was found for percentage of natural area cover and overweight, while significant negative correlation values were found between overweight and per capita natural area. This was identified particularly in the districts that had lower social conditions. On the other hand, the districts with the highest social conditions had the comparatively lowest levels of complete measles immunization. This study may facilitate public health work by identifying the urban areas in which the strengthening of health resources and actions should be prioritized and also calls for the inclusion of natural areas among the social health indicators included in intra-urban health inequality tools.

## 1. Introduction

### 1.1. Health Inequality Indicators

The rapid pace of urbanization places new demands on health care systems and on public health efforts. These demands include identifying and monitoring health inequalities to efficiently and equally distribute health efforts. One approach to this work is to study risk and resource factors and health determinants on a social and environmental level to assess and apply preventive interventions.

Health inequalities stem from social and socioeconomic health determinants, which are well recognized and broadly studied [1,2], although not always sufficiently approached [3]. These determinants reflect a person’s position in society, such as his or her education status, employment, income, and housing. The association between social conditions and health inequalities is evident with some biological causality, but without a direct measurable pathway, and the reasons for this relationship must be sought in individuals’ socio-economic contexts and living conditions. Social and health inequality is expected to grow with increasing urbanization and with climate change, thus affecting people’s chances to create healthy and prosperous lives from childhood and throughout life [4]. For example, increasing urbanization will result in a higher exposure to noise and air pollution [5], especially in deprived areas [6], causing increased mortality due to inflammatory and cardiovascular diseases in the affected populations [7,8,9]. From a life course perspective, this is particularly problematic because poor conditions during childhood and adolescence act as cumulative biological risk factors in the individual [10,11].

Reducing the socioeconomic inequalities that lead to the differences in health outcomes is a key concern for local and international authorities, including the Committee for Social Determinants of Health (CSDH) of the World Health Organization (WHO) [12] and steering guidelines such as the Health in all Policies (HiaP) and the Health 2020 framework. Efficient approaches to improving social conditions and social health determinants are necessary to reduce the preventable differences between various geographical regions’ and populations’ health outcomes. Social determinants of health are often amenable to change through policy, planning, and governance interventions, which have an indirect but substantial impact on public health [2].

To efficiently identify and prevent health inequalities, reliable indicators of socioeconomic conditions and indicators related to the availability of and access to health services are needed. These indicators can include information on income, government spending on health care, or access to safe environments. European statistics can be obtained from the WHO and the European Commission (e.g., the HEIDI data tool and EUROSTAT). Currently, many of these indicators are on a national or sub-national level.

There is often a strong correlation between different social indicators but also non-causal associations occur; for example, in Berlin, the number of dentists per 100,000 inhabitants is correlated to life expectancy at birth [13]. This clearly does not describe a causal relationship, but it does demonstrate a distributional pattern of health risks and resources. These types of patterns are likely to exist for many indicators.

In response to emerging intra-urban health inequalities, indicators or determinants of inequalities need to be identified at the urban district or sub-district level. Different tools for monitoring and localizing indicators within cities have been developed, such as the WHO tool Urban Health Equity Assessment and Response Tool (Urban HEART) [14]. The presence of intra-urban health inequalities demonstrates that a city’s average availability of health resources is not sufficiently informative; understanding a city’s spatial distribution and allocation in relation to different housing areas and livelihoods is crucial for achieving equal health opportunities. This is particularly important for addressing health inequalities among children, as they spend most of their time in their local neighborhood and are therefore likely to be uniquely affected by sub-regional conditions [15]. Children from less wealthy families in particular can be expected to spend much of their leisure time in their close neighborhood, as they have fewer opportunities to travel or visit other places or activities.

In general, children and adolescents in Europe today have better nutrition, health, and development than ever before. However, there are striking and increasing inequalities across the region, within countries, and within cities; for example, there is an over ten-fold difference in infant and child mortality rates across the 52 countries of the WHO European Region [16]. Another issue is the epidemic of childhood obesity, which is more prevalent in families from lower socioeconomic groups [2]. These inequalities necessitate a careful examination of children’s social and physical environments to ensure that healthy conditions are established where they are most needed.

### 1.2. Environmental Health Determinants

The health determinants map by Barton and Grant [17] places people at the heart and shows health determinants encompassing the ecosystem, the connection to climate change, biodiversity and human health while acknowledging social, economic and environmental aspects.

In this regard, environmental determinants have increasingly been recognized for their role in identifying distribution of health inequalities. Most often environmental determinants are risks, such as air pollution and noise, or lack of resources, such as poor sanitation and lack of clean water [14,18]. These determinants demonstrate the distributional inequality or equality of healthy environments [19,20,21] and the role of environmental deprivation in explaining health inequalities [18]. However, indicators of positive environmental resources are only rarely included among the existing health inequality indicators and tools. Although it is not particularly intended to identify health inequalities, the WHO uses access to urban green spaces as an environmental health indicator to monitor member countries’ fulfillment of the Parma Commitments: “to provide each child by 2020 with access (…) to green spaces in which to play and undertake physical activity” [22,23]. Although the WHO uses “access” to green space as indicator, in this paper we refer to the concept of availability rather than to the concept of accessibility because in the latter case, we argue that green spaces are often not fully “accessible” (for a methodological discussion see Kabisch et al. [24]).

Natural environments, which include urban green and blue spaces (parks, trees, gardens, lakes, rivers, etc.), are environmental health indicators of interest because they can provide a number of health opportunities, such as stress relief and physical activity [25,26], but are also beneficial for ecosystem regulation, such as reduced air temperature and pollution [27,28]. Based on statistical analyses, Maas et al. [29] and de Vries et al. [30] showed that a greener environment has a significant positive relation to perceived general health and also mental health of residents. More recent research has demonstrated a broad range of positive health outcomes that are associated with contact with natural environments, such as reduced mortality [31], improved course of pregnancy [32], cardiovascular health [33], and mental health outcomes [34,35] and reduced symptoms of depression [36,37]. Children’s cognitive, emotional, and motor development are all positively associated to exposure to nature [38,39,40,41]. In addition, street tree density and other urban greenery have been associated with less childhood obesity and increased physical activity [42,43,44,45]. Interestingly, many studies have demonstrated that health inequalities tend to decrease in greener areas [46,47] and that deprived groups seem to benefit the most from the positive health effects of nature [48,49]. Biological mechanistic explanations for these health benefits are not entirely elucidated, but experimental research suggests that both psychophysiological [50] and neurobiological [37] mechanisms may be at play.

Urban natural areas help improve children’s health when they are located either near their place of residence, along their daily route to school, or near their school (including school grounds). Therefore, in addition to a city’s general availability of natural areas, intra-urban “spatiality” is also important [51].

However, sufficient natural areas may not be available to all the population groups in a city. Because of biophysical and landscape prerequisites, natural areas and the quality of these spaces are often unequally distributed between groups of different socioeconomic status, age, and ethno-racial characteristics [52,53,54,55,56,57]. There may also be differences in how various groups use natural areas. Some studies have indicated that children in more deprived areas are less likely to be outdoors, partially because of the poorer quality of these areas compared to those in more wealthy areas, although the findings may not be totally conclusive as the amount of parks (though perceived as of lower quality) was higher in socio-economically deprived areas [55].

In addition to often having private gardens, households with a higher social status can be expected to compensate for a lack of natural areas in their direct housing environment by taking their children to participate in various activities in green environments outside the city. On the other hand, deprived and single-parent families are rarely able to afford these “green replacement” strategies [57].

The uneven distribution of natural areas may be due to a number of interrelated factors, including path dependency related to history, land use development, and park management and design [7,56]. Higher income households may have settled close to or around the green spaces in early urban history, which then established a pre-existing unequal distribution of green and prosperous environments. Accordingly, the development of natural areas in cities today and in the past may contribute to some of the complex socio-spatial inequality patterns that now exist in our cities.

### 1.3. Objectives

Based on the literature above, one could hypothesize that in addition to other already used indicators, such as socioeconomic status, the availability of urban natural areas may further facilitate the identification of health inequalities. The influence of environmental factors deserves more attention than it currently receives. Environmental factors have the potential to reduce socio-economic inequalities because their distribution is more easily influenced by urban planning. In addition, environmental factors will almost certainly become increasingly significant as urbanization and climate change increase, leading to more frequent heat waves, tropical nights, and flood risks. Various scenario studies and existing cases show that climate change most strongly affects those who are the most vulnerable, such as children and those who are socioeconomically deprived [58]. These findings demonstrate why environmental resources, such as green spaces for urban air temperature cooling [27,59], have an important role to play for improved health in vulnerable populations and thus might reduce health inequalities [46,47].

The aim of this study was to investigate the relationship between the spatial, intra-urban distribution of natural areas, such as green and water areas, and other known social health inequality indicators among children. We also wanted to explore whether the distribution of natural areas demonstrated a pattern that could be used to identify health inequalities on a sub-district level within a city, similar to other health determinants.

## 2. Materials and Methods

### 2.1. Study Area

Using one of the greenest cities in Europe—Berlin, the capital of Germany—as a case study, we studied the distribution of known health inequality indicators among 5- to 6-year-old children in Berlin’s sub-city districts.

**Figure 1:**
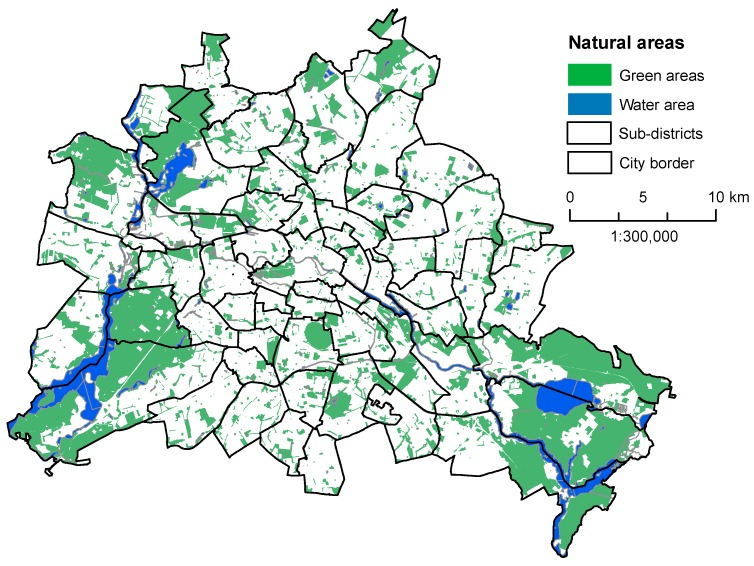
The case study, Berlin, and its distribution of green and water areas. Data are based on Berlin’s Environmental Atlas (Senate Department of Urban Development and the Environment, 2011).

Berlin is located in the lowlands of northern Germany, which is an area characterized by shallow river valleys and low-rise plateaus. The administrative boundaries of the city extend over a region of more than 89,000 ha. Nearly 40% of the city is composed of green and blue areas, including 14.5% public green space, 18.3% forest area, and 6.7% water area [60], but these spaces are very heterogeneously distributed across the city. Figure 1 shows their distribution throughout the whole city based on administrative city boundaries. The green and blue areas further expand behind the city border into the outer suburban or peri-urban areas. Some of these suburban areas have high shares of urban forest, while other areas purely consist of agricultural land (see Supplementary Material Figures S1 and S2).

Berlin’s population was 3,562,166 in 2014 and is expected to grow to 3.75 million over the next 15 years [61]. Thus, the population density in most districts will increase and pose further challenges to urban planners if they are to establish, maintain, and save their green areas.

### 2.2. Data

Available data on children’s health indicators were acquired from Berlin’s Senate Department for Health and Social Issues. The data are based on the medical check-ups of children (5 to 6 years old) in 2013, prior to school enrollment. In total, 30,427 children received a medical check-up (52% boys, 47% girls, 37.6% migration background) [62]. The best publicly available spatial data are on an anonymized, aggregated level of the 60 sub-districts of Berlin and include both the health outcomes and the social variables of the children and their families (for details see Table 1). Individual data on a finer spatial level were not available because of confidentiality regulations in Germany.

The health outcomes included overweight, dental problems, and deficits in language and viso-motoric development. The social variables reflected the participant’s social position, including the social status index of the parents (defined as educational attainment, graduation, and current employment status), the percentage of children living in single parent households, and the percentage of children with a non-German background. They also included two preventive variables: measles immunization coverage and participation in the preventive health check-up “U8” at the age of four. National health insurance offers nine preventive health check-ups, “U1 to U9”, to every child between birth and six years of age. The costs are covered, and there is no extra payment necessary from the parents. Dental health care checks are part of the preventive health check-ups. Regular dentist visits in kindergarten also occur every six months, and the national health insurance covers a number of early dental health care checks. Finally, variables related to the socio-environmental conditions of the child’s care, namely kindergarten attendance, having at least one smoking person in the household, and owning a television (TV), were included.

Local land-use data, stored in a Geographic Information System (GIS), were provided by the Berlin Senate Department of Urban Development and the Environment. Different environmental features can be categorized and related to various epidemiological factors, which are also stored in GIS-layers [63]. Land-use data came from Berlin’s Environmental Atlas project and reflected the composition of the city’s blocks. These data included population density and the percentage of areas that the city department defined as “simple residential,” or “einfache Wohnlage” in German. Specific criteria determine whether an area is classified as “simple” such as being less well maintained and having less affluent residences with low amounts of green space. In addition, availability of natural areas, including green and water areas (defined as a linear distance of a maximum of 300 m to a green or water space that is at least 2 ha), the total percentage of natural areas, and the per capita of natural area were included in the dataset. Both green and water areas are assumed to have positive health effects and in our analyses, and we combined theses spaces into one variable: “natural areas”.

The geographical delineation of sub-districts was based on a spatial hierarchy of Berlin called “living environment areas” (LEAs). The concept of LEAs was developed in 2006 and represents the basis for the urban planning, prognosis, observation, and administration of the city. The hierarchical structure contains three levels: 60 prognosis areas, 138 district regions, and 447 planning areas. In this paper, we analyzed the 60 prognosis areas for data availability and comparability reasons. For simplicity, these areas are referred to as sub-districts in the remaining text. On average, a prognosis area is approximately 15 km^2^ and has an approximate population of 55,000 people.

### 2.3. Statistical Analysis

We applied different methods to identify the most significant indicators of children’s health and health inequalities. These methods consisted of a bivariate correlation analysis, a factor analysis, and finally a cluster analysis to demonstrate the spatial distribution of possible relationships.

Following Schwarz [64] in her methodological approach, the linear correlation analyses were conducted with the health outcomes and health determinant variables before performing the factor analysis. The correlation analysis provided a first indication of the potential relationships between these variables. We used Spearman’s Rho as a correlation measure [19,64].

In the second step, a factor analysis was used to determine the minimal number of possible health inequality indicators to use in the following cluster analysis. Both health indicators and outcomes were included in the factor analysis because the primary objective was to identify the relationships between both sets of variables in the following cluster analysis. We chose the principal axis as the extraction method, which produces orthogonal (and therefore uncorrelated) factors, and a Varimax rotation was performed. The number of factors to be extracted was determined by the Eigenvalues of the factors. It was decided a priori that only factors with an Eigenvalue greater than one were to be extracted.

In the third step, a hierarchical cluster analysis was computed with the indicators identified by the factor analysis to demonstrate the spatial distribution and potential intra-urban patterns of health inequality indicators. The Ward procedure was applied with the squared Euclidian distance as the distance measure; this measure is often applied for these types of mixed social and land-use variable data [65]. The produced aggregation schedule and the dendrogram are provided in the Supplementary Material (Figure S3 and Table S1).

For the final cluster solution, ANOVA (Analysis of Variance) was calculated, including inter-cluster comparisons, to test if the indicators’ mean values within the clusters are significantly different between the variables included. All statistical analyses were conducted with SPSS 20.0 (IBM Corp., Armonk, NY, USA).

## 3. Results

### 3.1. Correlation Analysis

The bivariate Spearman correlations between health outcomes and the three other groups of variables—social, socio-environmental, and land use—are shown in Table 2. The correlation coefficients provided a preliminary indication of the relationships between health outcomes and possible determinants.

In the first group, i.e., the correlations between health outcomes and social variables including preventive factors, we found strong, significant values for nearly all the relationships. All the health-outcome variables showed a negative association with social status index. The higher the participants’ social status, the lower were their percentages of overweight, dental problems, and deficits in viso-motoric and language development. The variable “percentage of children with a non-German background” was strongly positively correlated with overweight, dental problems, and language-deficits. Regarding the preventive variables, positive correlations were found between receiving a complete measles immunization and health-outcome variables.

The correlations between the second group of variables—socio-environmental—and health outcomes also showed significant values. Attending kindergarten for at least two years was strongly negatively correlated with overweight, dental problems, and deficits in language development.

The third group of variables, land use, demonstrated several significant correlations to health outcomes. The strongest correlation was found between the percentage of addresses in “simple residential areas” and overweight, dental problems, and language-deficits. The percentage of natural area showed a significant, negative correlation with deficits in viso-motoric development, while natural area per capita demonstrated negative correlation values with the other three health-outcome variables. No significant correlations were found for the variable “availability of natural areas”.

The correlation analyses between social, socio-environmental and land-use variables revealed significant relationships, particularly between the percentage of addresses in “simple residential areas” and most of the social variables (see Table 3). Having a non-German background was positively correlated to living in dense districts and negatively correlated to per capita natural area. A significant negative correlation was found between availability of natural areas and social status index. A significant correlation was not found between natural area cover and the social health variables.

The correlation analyses between the social and socio-environmental variables (Table 4) identified a number of significant relationships. Children living in families with a higher social status had a higher prevalence of participation in U8 and kindergarten attendance, but a negative correlation was found between higher social status and complete measles immunization. High negative correlation values were also found between percentage of non-German households, percentage kindergarten attendance and participation in U8.

### 3.2. Factor Analysis

All the described variables were included in the factor analysis, as they all showed significant correlations. In total, 17 variables were included. The factors with Eigenvalues over one were extracted and explained 82.05% of the variance in the overall dataset. The first row in Table 5 summarizes the variance in the data that was explained by each factor. The communalities of all the indicators are shown in the last column, and they represent the amount of variance in a variable that is explained by all the factors. In Table 5, the factor loadings of all the indicators are also shown and sorted according to their values. The main loadings on factor one were health-outcome variables such as overweight or deficit in language development but also social and socio-environmental variables such as kindergarten attendance and social status. One land-use variable – simple residential area – was also highly loaded on factor 1.

The main loadings on factor two were related to the socio-environmental conditions of child care such as single parenthood, living in a household where at least one person smokes, and possession of a TV. The percentage of natural area cover, per capita natural area, and availability of natural area all loaded on factor three. Two variables loaded on factor four: population density and measles immunization, the latter showing the highest loading on this factor.

### 3.3. Cluster Analysis and Characterization of the Sub-Districts

A cluster analysis was used for the spatial characterization of sub-districts based on the results from the factor analysis. Following Schwarz [64], we used the variable with the highest factor loading per factor. Therefore, the four variables used for the cluster analysis of sub-districts were overweight, single-parent households, complete measles immunization, and natural area cover. We admit that using only the highest loading variable per factor in the cluster analysis will mask the accumulation of health problems in the same children (e.g., factor 1: overweight, dental problems, deficit in language) which is also strongly associated with non-German households and social status. However, our intention was to show a spatial distribution which is not the intrinsic intention of a cluster analysis but it can be used when the cases refer to a spatial class, in our case the sub-districts of Berlin.

**Figure 2:**
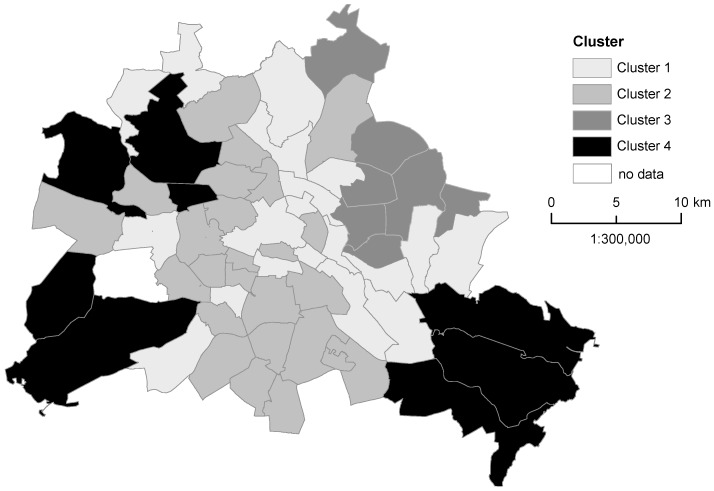
The results of the cluster analysis including the percentages of the variables overweight, single parent household, natural areas, and complete measles immunization.

Four significant clusters were identified. To characterize the sub-districts that belonged to each cluster, standard descriptive statistics are shown in Table 6, including the mean value, standard deviation and the number of sub-districts within the cluster. Table 6 further illustrates the results of the ANOVA, including inter-cluster comparisons of the indicators’ mean values. The results indicated significantly different mean values between all the included variables. Figure 2 shows the spatial distribution of the sub-districts according to their clustering. Supplementary material Figure S4 additionally demonstrates the spatial representation of the four indicators from the factor analysis on a sub-district level.

Cluster 1 contained 19 sub-districts. They were predominantly situated in the inner part of the city but were also found in other parts of the city. Cluster one had the lowest percentage of overweight children (5.79%), single-parent households (19.48%), and children with complete measles immunization (88.59%) compared to all the other cluster mean values. The mean natural area cover was 20.21%, which was the second highest among the clusters. Cluster 2 contained 25 sub-districts and was characterized by the highest mean value of children with overweight (11.48%), the lowest share of natural area coverage (15.16%), and a comparatively high percentage of complete measles immunization (92.89%). In Cluster 3 (seven sub-districts in the north-eastern part of the city), the percentage of children with overweight was near the city average (8.41%), and nearly 40% of the children lived in single parent households. As in Cluster 2, the share of natural area coverage was relatively low (15.30%). The mean value of the percentage of children with complete measles immunization was the highest in cluster 3 (93.26%). Finally, the eight sub-districts of Cluster 4 were characterized by the highest percentage of natural area coverage (55.54%), and the percentage of children with overweight (7.58%) and complete measles immunization (90.19%) were both below the city’s average. The percentage of single parent households (23.68%) was around the city average.

## 4. Discussion

This study demonstrates a socio-spatial distribution of natural areas in relation to health-inequality indicators of children in Berlin. Four potential dimensions of health inequality—overweight, single parent household, natural area cover, and measles immunization—were distributed throughout the city according to a certain spatial pattern. Sub-districts with a relatively large proportion of natural area cover also had low percentages of children being overweight or living in single parent households. This supports the hypothesis that the distribution of natural area cover may spatially overlap with the distribution of other, regularly used indicators of intra-urban health inequalities. Another finding was that the sub-districts with indicators that correlated with higher social status had a comparatively low percentage of children with full measles immunization.

This study was not conclusive regarding any causalities between natural area cover and health inequality, and the results only partly supported our initial hypothesis. The green and water areas only correlated with some of the social variables in the bivariate correlation analysis. Nevertheless, all environmental variables explained about 10% of the variance in the factor analysis assumed to correspond to health inequality. However, as the natural area cover did show a clear spatial pattern that overlapped with social patterns, the results reflect the need for further investigation of “green” inequality indicators, especially in other cities where green spaces may be less abundant than in Berlin. This could potentially promote policy interventions and governance activities for developing healthy, natural areas in areas where they are most needed, particularly for children. Urban planning efforts should take these findings into consideration. Furthermore, these findings may also help avoid the so-called “green paradox” or “eco-gentrification” [66,67], which suggests that, for example, higher housing prices in appealing areas with nearby high quality natural areas can lead to the displacement of vulnerable residents for whom these spaces would be the most beneficial [48,49]. For residents with smaller financial means, natural areas could potentially function as a complementary health resource, counteracting some of the socially determined health inequalities. This assumption is based on an extensive amount of research demonstrating the positive health effects of natural areas [25] as well as reduced health inequalities [46].

If the lack of natural area cover is an indicator of health inequalities, then the natural areas in cities may be suitable for inclusion in intra-urban health inequality tools, such as the Urban HEART [14]. By including natural areas, health inequality tools could potentially be refined and improved by adding a preventive environmental dimension to the social one, which could contribute to steering appropriate public health and planning actions. With increasing global urbanization and higher pressure and competition regarding urban land use worldwide, these findings may be relevant for not only European cities, but can be expected to become even more important worldwide.

In our study, we observed a significant bi-variate correlation between natural area cover and viso-motoric development. In the cluster analysis, a high percentage of natural area cover was also one of the main factors defining the clusters that included low levels of overweight and relatively low percentages of single parent households. Availability of natural areas was not significantly associated with the included health outcomes, but it was negatively correlated with social status index and positively correlated with smoking in the household and TV possession. The positive correlation between natural area cover and smoking in the household and TV possession may be explained by the general high coverage of natural area in Berlin, which would make the differentiation between areas less distinct. Even in sub-districts with higher shares of “simple residential areas,” such as those with prefabricated large housing estates (e.g., sub-districts Marzahn), large amounts of natural areas can still be found, although they may differ in quality. The quality of a natural area can be defined through cleanliness or through diversity, for example a natural area that provides different amenity features including large trees providing shade, specific sports grounds and enough benches. Natural areas in Berlin are generally well maintained by the district’s green space departments, and in terms of cleanliness, the quality is high almost everywhere. However, the diversity of amenity features does differ, resulting in potential non-use of some spaces. Some spaces are provided with lawns that are used for sports, but they lack numerous benches or large trees (e.g., the Tempelhofer Feld—the former city airport). Specific population groups use these types of spaces less frequently (e.g., elderly people, see an extensive discussion by authors [20,68]). The quality and amenities of natural areas were not included in our land-use data, but they should be considered in future studies. For example, future studies could benefit from including audits of greenspace conditions and facilities [69].

The natural area per capita was correlated with several health outcomes but was only correlated with one social indicator, non-German background. These somewhat mixed results demonstrate the inherent complexity in correlations between social and environmental factors and health outcomes including inequalities. Socially determined health outcomes are usually multifactorial and escape simple linear relationships. This complicates epidemiological analyses, as it is difficult to demonstrate the strengths of correlations, and univariate or linear explanations of the complex impact on health cannot be expected. This calls for alternative statistical approaches, such as exploring spatial distribution, rather than examining linear correlations to identify direct causality. By demonstrating the spatial patterns of various indicators, in our case both environmental and social, a contextual interpretation may better inform us about what patterns contribute to health problems and how combined efforts could be achieved to reduce inequalities. One suggestion could be to modify the green and blue infrastructure on the one hand and promote more social integration on the other.

This paper also prompts further discussion about the adequacy of existing urban green or natural area indicators. Either availability [23] or total green space coverage is often used to define green space potential [70]. Our results are not conclusive as to whether natural area cover, natural area per capita, or availability of natural area is the most appropriate metric to use to indicate health and inequalities. All three of these variables defined a common factor in the analysis, but in the correlation analysis, their relationships to other indicators varied. The per capita value correlated with the non-German background variable and the accessibility to social status index. In general, this would suggest that a green or natural area indicator for health should incorporate several aspects of natural area including the percentage of coverage, per capita, and availability values, and if possible the quality of the area as well. Most importantly, the spatial distribution of natural area indicators should be carefully considered and incorporated into decisions regarding efficient resource allocation with a particular focus on the districts that are currently less green (or blue) or are deprived.

Many availability threshold values seem to adhere to the common practice of using a maximum 300 m linear distance to a green space of at least 1–2 ha [23,71]. However, these thresholds do not consider how many people actually live within the recommended distance and therefore do not take into account the pressure on the area and the risk for crowding and over-use [24]. This suggests that including a per capita value into natural area availability indicators could be valuable. For example, in Berlin, green spaces and waters are distributed throughout the whole city, and this can result in good overall availability values despite low per capita values in certain areas. The threshold used for defining availability in our study (maximum 300 m linear distance to a green space of minimum 2 ha) may not have been the most appropriate for identifying differences on a sub-district level. It is also plausible that for children, smaller green spaces and patches that they can easily go to for play and physical activity may be beneficial, particularly if they are nearest in vicinity to their home. A 300 m linear distance is often longer in reality, as the linear measure does not consider the actual walking route, including larger roads and other physical barriers. Therefore, the 300 m threshold may be less relevant for children. In addition, it has been found that barriers that increase the walking distance are more frequent in areas of social deprivation [57]. Integrating a street network analysis may improve the results further. In this analysis, unfortunately, road data could not be included as they appear as line elements, which are too dominant in the GIS calculation at the scale level we used. Of course, they should be included when calculations are done at a finer scale level, such as a neighborhood scale and may then potentially affect the results at this scale.

One of the social, preventive indicators that did not follow the perhaps expected pattern was measles immunization. The sub-district with the lowest rate of immunization was also the wealthiest area, which presented the highest social status values and a comparatively high percentage of natural area cover. This finding is interesting considering the fact that Berlin has been facing a severe outbreak of measles since 2014, which is at least partly explained by the increasing numbers of children in districts with higher social conditions who are not being vaccinated. Similar results of a negative relationship between social status and full measles immunization were found in a study in Munich, Germany, by Koller and Mielck [15].

The seemingly socially determined decline in vaccination rates may be due to previous media warnings that falsely claimed an increased risk of autism as a side effect of measles-mumps-rubella (MMR)-vaccination; these false warnings have resulted in declining immunization rates in many European countries [72,73]. This has had the serious and truly unfortunate effect that we are now seeing a rising prevalence of measles and other vaccine-preventable diseases [74]. It is plausible that parents with higher education levels have better access to media and thus become more aware of and attentive to such warnings, consequently denying their children the benefits of immunization out of good, though misguided, intentions. Many studies on the negative effects of vaccination have a spurious or ambiguous scientific base [75,76]; however, this is not always clear in the media reports, which could explain why these findings may be perceived as scientifically-based advice. This decrease in immunization rates has been occurring despite solid evidence that the potentially adverse effects of measles vaccination are negligible in comparison to the indisputable positive effect on reduced child mortality [77]. Not one high quality study has demonstrated any association between MMR-vaccination and autism or any other neurodevelopmental disorders [78,79].

Although it has previously been speculated that migrants are more likely to accept recommendations from health care professionals [15], this claim was not supported by our results regarding U8 attendance, nor by others’ findings on participation in health check-ups, which are often lower in deprived areas [80].

### Limitations

One of the limitations of this study was the non-generalizability, as the results were based on a single case-study, Berlin. However, the results did indicate that the hypothesized correlation between natural areas and other social health determinants existed to some extent and that those factors both displayed a certain intra-urban spatial pattern. These findings warrant further studies in other locations.

Results are further based on bivariate correlation analyses which might have led to the inconsistency in causality. To address this, supplementary Tables S2–S4 include a hierarchical multivariate regression in which we treated social and social-environmental variables as confounders and the land use variables as predictors to predict health outcome. We found a similar inconsistency in causality with the regression models as in the previous analyses. Percentage natural area was not significantly adding to the explanation of the variance in the model predicting overweight (Table S2). However, natural area cover and per capita natural area significantly contribute to the explanation of total variance in the models predicting deficits in viso-motoric (Table S3) and deficits in language development (Table S4).

Our study also relied on the available data, and we may have missed other important health inequality determinants. We cannot say whether additional indicators would have weakened or strengthened the influence of natural areas. Examples of indicators that have been used in other tools for health inequality assessments are government spending on health and urban planning and access to safe water and sanitation. However, many indicator tools rely on publicly available data, which ensures replicability and monitoring opportunities. From a European perspective, the indicators available for our study should be fairly relevant, and the results could be used to discuss healthy environmental planning on a sub-district level in Berlin. This should encourage further testing and could potentially result in the development of a European health equity assessment tool for children that includes a dimension of natural areas.

The available data on health outcomes among children in Berlin only included overweight, dental health, and language and viso-motoric development. To further advance this field and to develop a valid tool, the correlations between natural area and other health outcomes, such as diabetes, road traffic injuries, and stress-related disorders, should be explored. A further limitation of the presented analyses may be that we have not dealt with spatial autocorrelation. In further statistical testings x- and y-coordinates of sub-district centroids could be included.

We must also acknowledge the limitation that our analyses were based on aggregated data. This may have influenced the results, as the distribution of health and inequality indicators vary within each sub-district. This limitation must be considered when interpreting the results.

The land use data were from 2011, and updated data should be used for future analyses as soon as they are available. We also included all the green spaces and parks in the analysis, but some of them charge an entrance fee and are not publicly available to all the residents.

In this study, we combined data on green and blue spaces into a “natural area” variable, as recent research has demonstrated that blue spaces have similar health benefits as green spaces [81,82]. However, this combined variable has not been tested previously, and the blue spaces of Berlin, not being a coastal city, may not be of the same restorative character as in some other cities because they are, for the most part, not easily accessible to children. We also tested the land cover categories individually and the differences were not significant and small as compared to the combined variable.

Another combined variable in our analysis was the “social status index”. This variable included parents’ educational attainment, graduation, and current employment status. It has been used in other measures of socioeconomic situation in Germany, but it may be too crude in terms of identifying and localizing health inequalities and their relationship to environmental features.

Finally, we acknowledge that we are aware of possible multi-collinearity of the variables for which a hierarchical cluster analysis should not be used. However, we could not identify any high collinearity among the variables. We did identify correlation values on low levels only between the variables overweight and single parent households, overweight and complete measles immunization and single parent households and measles immunization. All not extending values of 0.35.

## 5. Conclusions

In this paper, we analyzed intra-urban relationships between children’s social health determinants and outcomes and natural areas on a sub-district level in the city of Berlin, Germany. We identified that a lower percentage of natural area cover was correlated with deficits in viso-motoric development of the children, as well as areas with lower natural area per capita had significantly higher values of childhood overweight. This was found particularly in the districts that are characterized by lower mean income and less favorable social conditions such as the inner city districts (e.g., Wedding or Neukölln) with high share of families with immigration background as well as in Marzahn, which is a well-known prefabricated large housing estate from socialist times where low income groups and single parent families cumulate. Thus, the health state mirrors typical social patterns of the city.

Our study further confirms that there is a certain socio-spatial distribution of natural areas in Berlin. This may contribute to and facilitate public health work by identifying areas where the strengthening of health resources and actions should be prioritized. Eventually, natural areas may be added to social health indicators in intra-urban health inequality tools. In addition, the results from the study may be useful for more efficient and needs-based urban green space planning and environmental management. Similar results have previously been found by for example Byrne and Sipe [83]. Through policy actions that are aimed at providing more and improved natural areas to deprived areas while consciously avoiding “green gentrification”, so-called “upstream” prevention could be achieved. Instead of relying on top-down interventions, such as bans and education, the environment would be inherently healthier (providing opportunities for physical activity, recreation, social interactions, and improved air quality), and people’s capacity to cope with difficult living conditions may be supported.

However, before implementation of the results, the causality of relationships should be further investigated, and the relationship between social and environmental factors and health inequalities should be scrutinized in various settings, contexts, and countries. Specific factors to be explored in future studies include the impact of different measurements for assessing natural areas and their availability as well as social health indicators’ potentially inverse relationship to spatial health inequality patterns. In our study, for example, this inverse relationship was found for the measles immunization variable.

In addition, we must be aware of the limited resources for extensive green establishment in today’s cities. Therefore, innovative solutions, such as opening currently private spaces or refurbishing less well maintained urban greyfields or wildlands, must be encouraged.

## Figures and Tables

**Table 1:** Description of the variables used in analyses.

Variables	Description	Year	Data Source
**Health-outcome variables**			
Overweight (%)	Percentage of children overweight as defined by the Body-Mass-Index, BMI (thresholds are defined monthly by Kromeyer-Hausschild)	2013	SDHS
Dental problems (%)	Percentage of children with dental problems	2013	SDHS
Deficits in viso-motoric development (%)	Percentage of children with impaired fine motor ability	2013	SDHS
Deficits in language development (%)	Percentage of children with deficits in language development	2013	SDHS
**Social variables**			
Social status index	Median index (0–18) representing the status of parents based on school education, employment education and employment status: 0–8 low social status, 9–15 medium social status, 16–18 high social status	2013	SDHS
Single parent household (%)	Percentage of children living in single parent households	2013	SDHS
Non-German (%)	Percentage of children with background other than German	2013	SDHS
Complete measles immunization (%)	Percentage of children with at least two doses (considered complete) of measles vaccination	2013	SDHS
Participating in U8 (%)	Percentage of children that participated in a preventive health check-up at the age of 4, i.e., U8	2013	SDHS
**Variables of social-environmental conditions of child care**			
Kindergarten attendance (%)	Percentage of children enrolled in kindergarten for at least 2 years	2013	SDHS
Smokers (%)	Percentage of children with at least one smoking person in household	2013	SDHS
Own TV (%)	Percentage of children with their own TV	2013	SDHS
**Land use variables**			
Simple residential area (%)	Percentage of addresses situated in an area classified as a “simple residential area.” These areas are very dense, highly contained and have low to no green space. The streets and building facades are mostly not well maintained.	2010	SDUDE
Natural area (%)	Percentage of green and water areas in the sub-districts in relation to the total sub-district area. Green areas include forest areas, urban green and parks, cemeteries and allotment gardens. Water areas include all the water bodies such as lakes, rivers and canals.	2011	
Per capita natural area (m^2^/inhabitant)	Natural area (m^2^)/total number of inhabitants in the sub-district	2011	SDUDE
Availability of natural area (%)	Percentage of inhabitants living a maximum of 300 m distance away from a natural area (green area of min. 2 ha)	2011	SDUDE
Population density (inhabitants/km^2^)	Total number of inhabitants in 2014/total area of the sub-districts (km^2^)	2014	SDUDE, Dep. for Statistics BBR

**Table 2:** Spearman correlation between health-outcome variables and health determinants.

	Health Outcome	Overweight (%)	Dental Problems (%)	Deficits in Viso-Motoric Development (%)	Deficits in Language Development (%)
Health Determinants					
**Social variables**					
Social status index		**−0.780** **	**−0.786** **	**−0.660** **	**−0.822** **
Non-German (%)		**0.809** **	**0.754** **	**0.334** **	**0.779** **
Single parent household (%)		**0.333** **	**0.475** **	**0.415** **	**0.365** **
Complete measles immunization (%)		**0.354** **	**0.394** **	**0.467** **	**0.470** **
Participating in U8 (%)		**−0.525** **	**−0.507** **	**−0.356** **	**−0.506** **
**Socio-environmental variables**					
Kindergarten attendance (%)		**−0.780** **	**−0.821** **	**−0.585** **	−0.873 **
Smokers in family (%)		**0.658** **	**0.686** **	**0.630** **	**0.685** **
Own TV (%)		**0.689** **	**0.728** **	**0.747** **	**0.754** **
**Land use variables**					
Simple residential area (%)		**0.520** **	**0.525** **	**0.444** **	**0.538** **
Natural area (%)		−0.149	−0.172	**−0.260** *	−0.248
Per capita natural area (m²/inhabitants)		**−0.322** **	**−0.334** **	−0.209	**−0.332** **
Inhabitants with availability of natural area (%)		0.105	0.102	0.116	0.106
Population density (inhabitants/km²)		**0.369** **	**0.393** **	0.168	**0.342** **

* *p* < 0.5; ** *p* < 0.01, significant correlations in bold.

**Table 3:** Spearman correlation between social, socio-environmental and land use variables.

	Social & Socio-Environ	Social Status Index	Non-German (%)	Single Parent Household (%)	Complete Measles Immunezation (%)	Participating in U8 (%)	Kindergarten Attendance (%)	Smokers (%)	Own TV (%)
Land-Use Variables									
Simple residential area (%)		−0.594 **	0.478 **	0.258 *	0.250	−0.566 **	−0.358 **	0.582 **	0.489 **
Natural area (%)		−0.02	−0.189	0.073	−0.095	0.002	0.128	0.019	−0.017
Per capita natural area (m²/inh.)		0.034	−0.462 **	0.013	0.139	0.209	0.232	−0.030	0.004
Availability of natural areas (%)		−0.316 *	0.006	0.181	−0.036	−0.175	−0.181	0.427 **	0.270 *
Population density (inh./km²)		−0.076	0.555 **	0.049	−0.228	−0.314 *	−0.267 *	0.089	0.013

* *p* < 0.5; ** *p* < 0.01, significant correlations in bold.

**Table 4:** Spearman correlation between social and socio-environmental variables.

Social & Socio-Environ. Variables	Social Status Index	Non-German (%)	Single Parent Household (%)	Complete Measles Immunization (%)	Participating in U8 (%)	Kindergarten Attendance (%)	Smokers (%)
Non-German (%)	−0.551 **						
Single parent household (%)	−0.576 **	0.132					
Complete measles immunization (%)	−0.551 **	0.123	0.342 **				
Participation in U8 (%)	0.542 **	−0.441 **	−0.354 **	0.010			
Kindergarten attendance (%)	0.742 **	−0.743 **	−0.372 **	−0.400 **	0.357 **		
Smokers (%)	−0.883 **	0.395 **	0.748 **	0.422 **	−0.550 **	−0.593 **	
Own TV (%)	−0.898 **	0.381 **	0.714 **	0.578 **	−0.526 **	−0.695 **	0.886 **

* *p* < 0.5; ** *p* < 0.01, significant correlations in bold.

**Table 5:** Factors extracted by factor analysis using all the health determinant and health outcome variables.

Variables	Factor (% of Variance)				Communalities
	I (48.97)	II (16.84)	III (9.67)	IV (6.57)	
Overweight (%)	0.919				0.891
Non-German (%)	0.915				0.898
Deficit in language (%)	0.907				0.954
Dental problems (%)	0.854				0.860
Kindergarten attendance (%)	−0.847				0.825
Social status index	−0.829				0.910
U8 participation (%)	−0.628				0.731
Simple residential area (%)	0.616				0.620
Single parent household (%)		0.907			0.829
Smoker in family (%)		0.820			0.904
Own TV (%)		0.776			0.947
Deficits in viso-motoric development (%)		0.648			0.667
Natural area (%)			0.924		0.876
Per capita natural area (m²/inhabitant)			0.866		0.813
Availability of natural area (%)			0.633		0.543
Complete measles immunization (%)				0.821	0.862
Population density (inhabitants/km²)				−0.609	0.822

**Table 6:** One-way analysis of variance (ANOVA) and the mean values of the clusters of Berlin sub-districts.

Cluster Indicators	Cluster					Total City
	ANOVA F (df, *p*-values)	1	2	3	4	
		M (SD) Cluster 1	M (SD) Cluster 2	M (SD) Cluster 3	M (SD) Cluster 4	
Overweight (%)	12.69 (3; 0.000)	5.79 (2.41)	11.48 (3.72)	8.41 (2.22)	7.58 (2.92)	8.76 (3.92)
Single parent household (%)	20.13 (3; 0.000)	19.48 (6.01)	23.48 (4.07)	39.89 (5.55)	23.68 (10.18)	24.17 (8.44)
Natural area (%)	51.84 (3; 0.000)	20.21 (7.29)	15.16 (7.16)	15.30 (9.39)	55.54 (12.03)	22.28 (15.68)
Complete measles immunization (%)	7.17 (3; 0.000)	88.59 (4.49)	92.89 (2.13)	93.26 (1.56)	90.19 (4.14)	91.18 (3.83)
N (districts)		19	25	7	8	59

## Supplementary Materials

The following are available online at www.mdpi.com/1660-4601/13/8/783/s1, Figure S1: Land cover of Berlin’s administrative city area and within a 15 km buffer around the city border. Data source: Urban Atlas 2006 (EEA, http://www.eea.europa.eu/data-and-maps/data/urban-atlas), Figure S2: Different green spaces in Berlin (from top left to bottom right): The Tempelhofer Feld, the former city airport which closed in 2008 and opened for public use in 2010 is situated only 5 km south of the city centre (Photo by author); tree desk—the area on the ground surrounding trees is planted by local residents all over the city area (Photo by author); the Gleisdreieck park was a former railway brownfield and is now a highly diverse area including parts for recreation, playgrounds for children or urban wilderness areas (Photo by author); the Rosengarten is an intercultural garden in the inner city (Photo by author); another part of the Gleisdreieck with lawns served for recreation (Photo by author); the Grunewald—an urban forest in the south-western part of the city (Photo by N. Larondelle); park around the Castle Charlottenburg—the oldest park in the city; the Görlitzer Park, a former railway area, only three kilometers southeast of the city centre (Photo by N. Larondelle), Figure S3: Cluster analysis—dendrogram, Figure S4: Distribution of the sub-districts according to the cluster variables. Table S1 Cluster analysis—Agglomeration schedule. Table S2: Outputs of hierarchical multivariate regression models on influencing factors of children overweight (%), Table S3: Outputs (Beta and *p*-values) of hierarchical multivariate regression models on influencing factors of children deficit in viso-motoric development (%), Table S4: Outputs (Beta and *p*-values) of hierarchical multivariate regression models on influencing factors of children deficit in language development (%).
