# Supplementary material for: Adding Natural Areas to Social Indicators of Intra-Urban Health Inequalities among Children: A Case Study from Berlin, Germany
Source: Int J Environ Res Public Health. 2016 Aug 4;13(8):783. doi: 10.3390/ijerph13080783 (PMC4997469; doi:10.3390/ijerph13080783)
Supplement: Supplementary file 1 [file ijerph-13-00783-s001.pdf]

Supplementary Materials: Adding Natural Areas to Social Indicators of Intra-Urban Health Inequalities among Children: A Case Study from Berlin, Germany

Nadja Kabisch , Dagmar Haase and Matilda Annerstedt van den Bosch

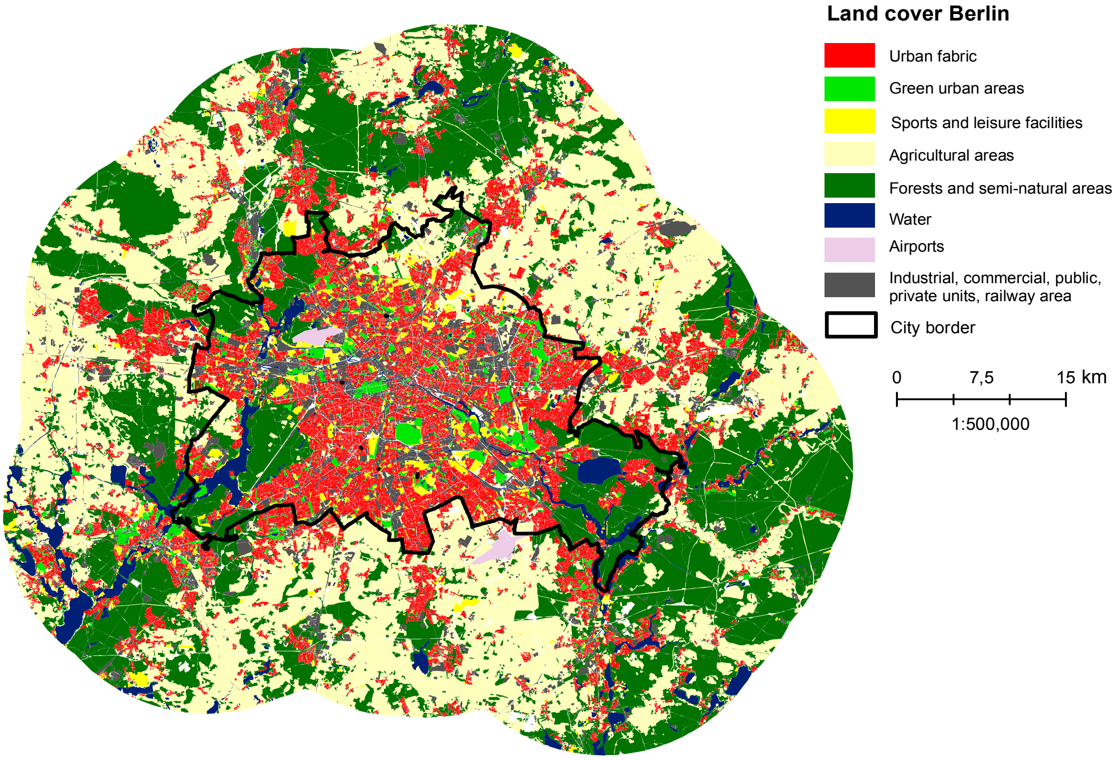

Figure S1. Land cover of Berlin's administrative city area and within a 15 km buffer around the city border. Data source: Urban Atlas 2006 [1].

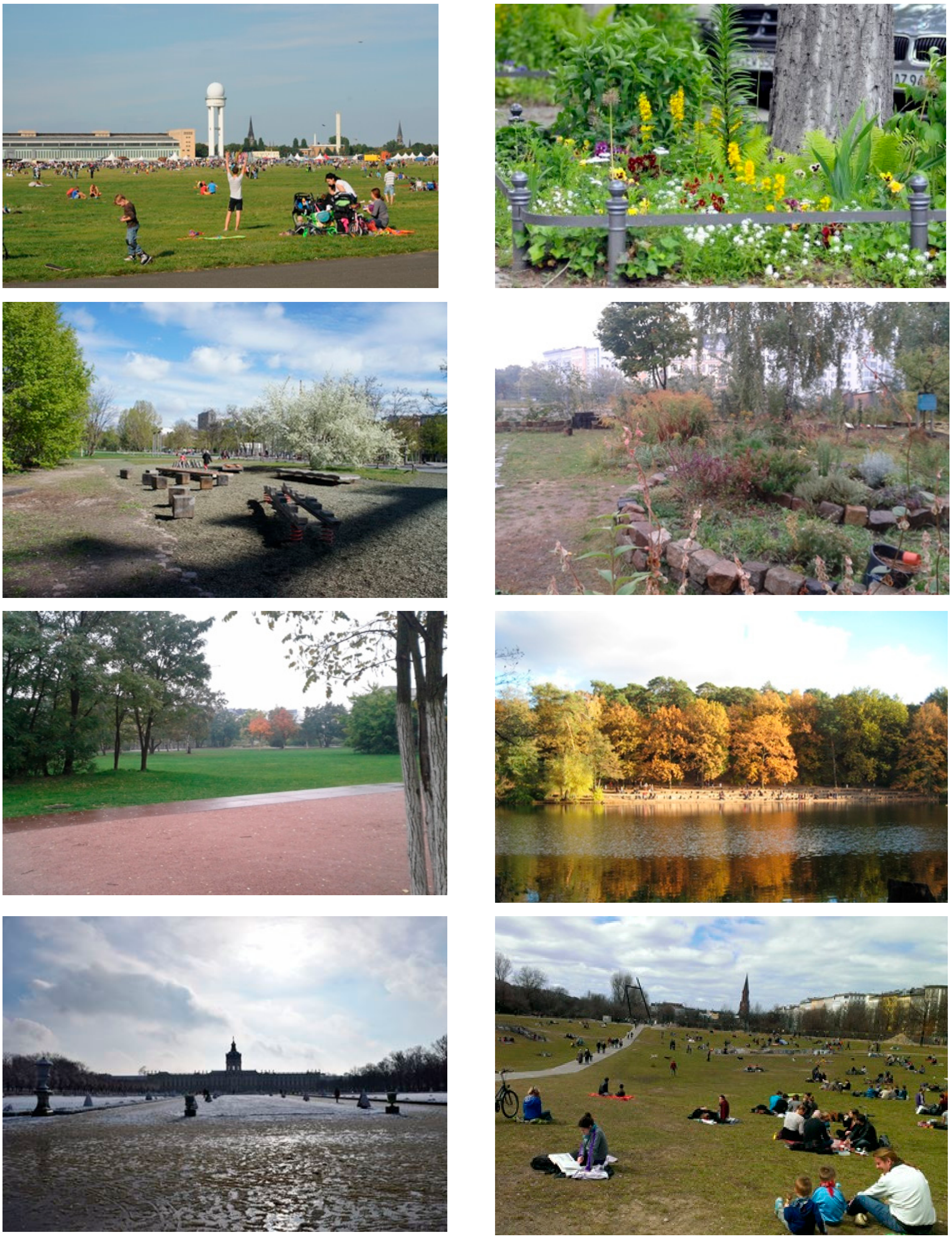

Figure S2. Different green spaces in Berlin (from top left to bottom right): The Tempelhofer Feld, the former city airport which closed in 2008 and opened for public use in 2010 is situated only 5 km south of the city centre (Photo by author); tree desk—the area on the ground surrounding trees is planted by local residents all over the city area (Photo by author); the Gleisdreieck park was a former railway brownfield and is now a highly diverse area including parts for recreation, playgrounds for children or urban wilderness areas (Photo by author); the Rosengarten is an intercultural garden in the inner city (Photo by author); another part of the Gleisdreieck with lawns served for recreation (Photo by author); the Grunewald—an urban forest in the south-western part of the city (Photo by N. Larondelle); park around the Castle Charlottenburg—the oldest park in the city; the Görlitzer Park, a former railway area, only three kilometers southeast of the city centre (Photo by N. Larondelle).

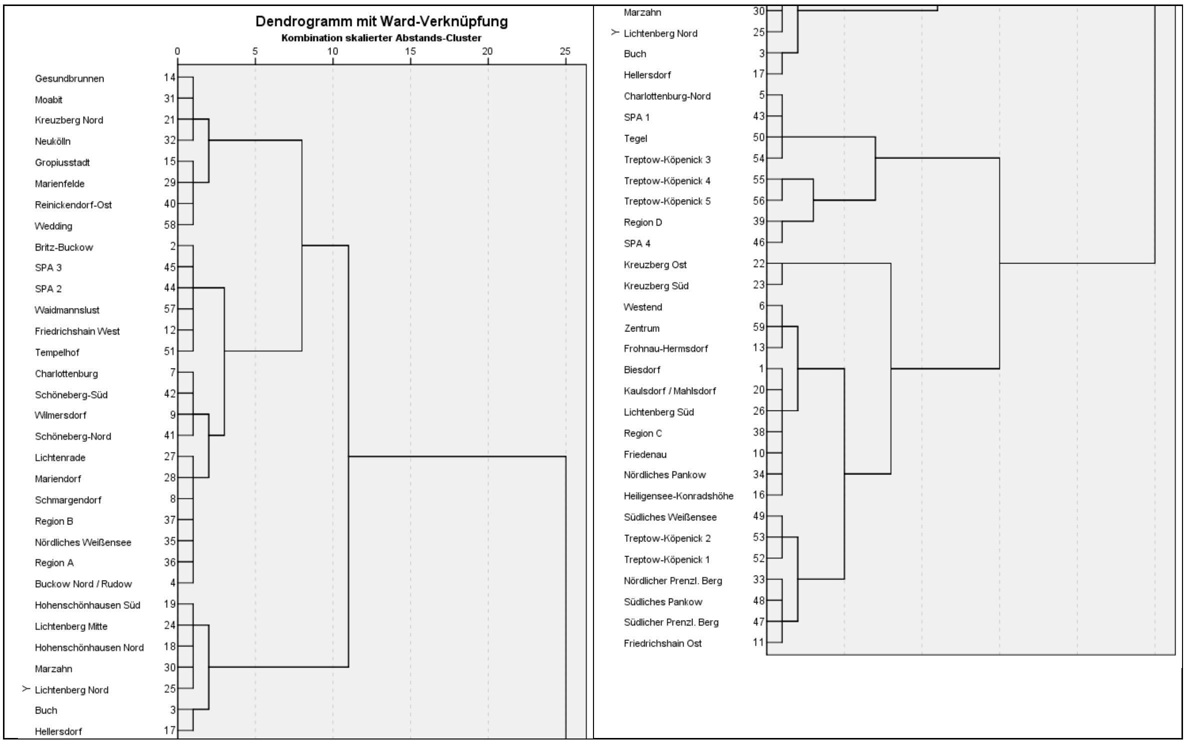

Figure S3. Cluster analysis dendrogram (upper part left, lower part right).

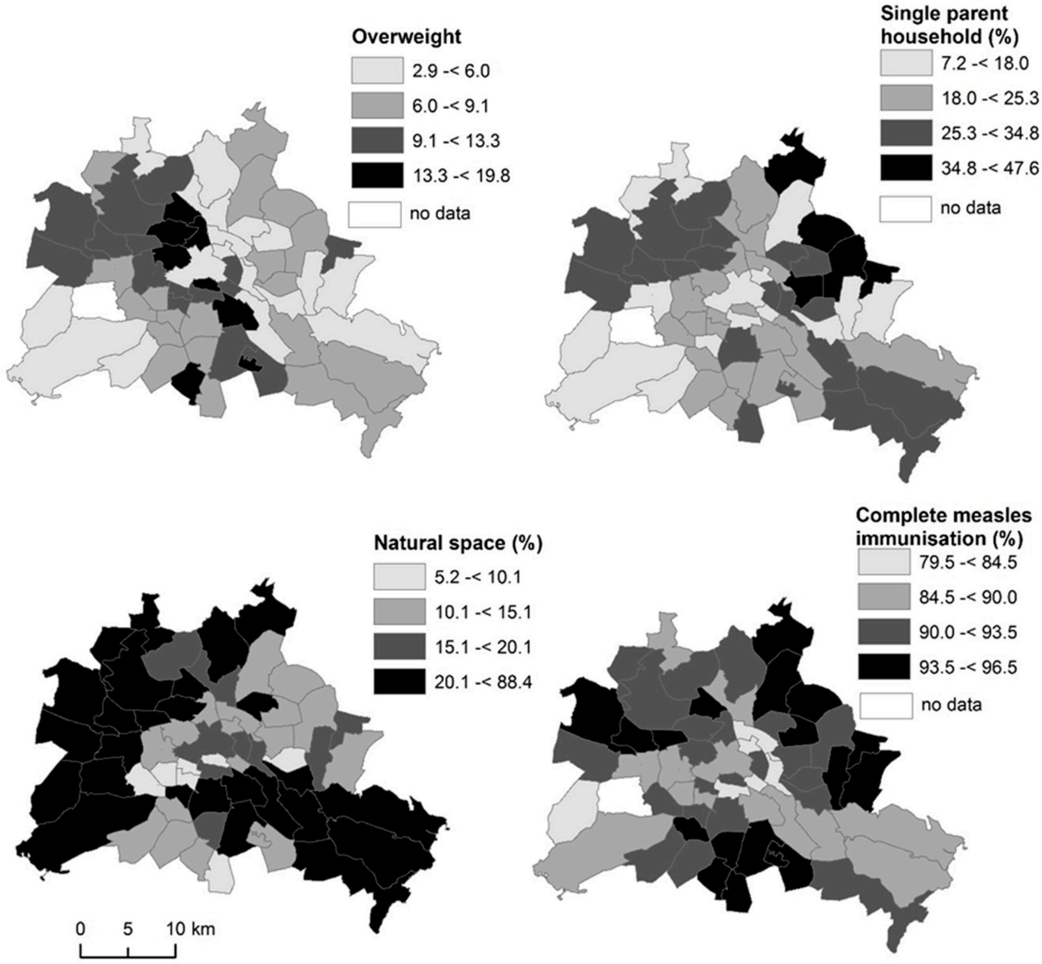

Figure S4. Distribution of the sub-districts according to the cluster variables.

Table S1. Agglomeration schedule as SPSS generated result from the hierarchical cluster analysis.

Stage	Cluster Combined		Coefficients	Stage Cluster First Appears		Next Stage
	Cluster 1	Cluster 2		Cluster 1	Cluster 2	
1	14	31	0.018	0	0	21
2	44	57	0.081	0	0	3
3	12	44	0.165	0	2	24
4	7	42	0.259	0	0	9
5	18	30	0.381	0	0	31
6	6	59	0.523	0	0	19
7	5	43	0.671	0	0	29
8	35	36	0.831	0	0	20
9	7	9	0.994	4	0	28
10	15	29	1.162	0	0	34
11	8	37	1.333	0	0	27
12	26	38	1.540	0	0	26
13	1	20	1.761	0	0	43
14	10	34	2.031	0	0	26
15	27	28	2.349	0	0	39
16	49	53	2.681	0	0	32
17	2	45	3.061	0	0	37
18	22	23	3.450	0	0	54
19	6	13	3.845	6	0	46
20	4	35	4.260	0	8	27
21	14	21	4.679	1	0	38
22	19	24	5.133	0	0	40
23	55	56	5.598	0	0	51
24	12	51	6.114	3	0	37
25	33	48	6.687	0	0	33
26	10	26	7.265	14	12	36
27	4	8	7.849	20	11	39
28	7	41	8.490	9	0	48
29	5	50	9.169	7	0	35
30	40	58	9.860	0	0	34
31	18	25	10.554	5	0	40
32	49	52	11.281	16	0	49
33	33	47	12.065	25	0	41
34	15	40	13.255	10	30	47
35	5	54	14.470	29	0	53
36	10	16	15.686	26	0	43
37	2	12	16.949	17	24	50
38	14	32	18.253	21	0	47
39	4	27	19.561	27	15	48
40	18	19	21.123	31	22	45
41	11	33	22.739	0	33	49
42	3	17	24.477	0	0	45
43	1	10	26.389	13	36	46
44	39	46	28.541	0	0	51
45	3	18	31.766	42	40	56
46	1	6	35.005	43	19	52
47	14	15	38.580	38	34	55
48	4	7	42.305	39	28	50
49	11	49	46.797	41	32	52
50	2	4	51.829	37	48	55
51	39	55	58.178	44	23	53
52	1	11	69.472	46	49	54
53	5	39	84.865	35	51	57
54	1	22	101.043	52	18	57
55	2	14	118.758	50	47	56
56	2	3	142.836	55	45	58
57	1	5	174.954	54	53	58
58	1	2	232.000	57	56	0

Table S2. Outputs of hierarchical multivariate regression models on influencing factors of children overweight (%).

	β_1 (Social Index)	β_2 (Non-German %)	β_3 (Single Parent Household %)	β_4 (Kindergarten Attendance %)	β_5 (Natural Area %)	β_6 (Access Natural Area %)	β_7 (Per Capita Natural Area %)	R^2-Adjusted (%)
Model 1	**−0.46**	**0.50**	−0.01	−0.06				84.6
p values	0.000	0.000	0.908	0.534				
Model 2	**−0.46**	**0.51**	0.00	−0.09	−0.15	−0.05	0.18	84.8
p values	0.000	0.000	0.984	0.358	0.154	0.431	0.111	

Note: Significant coefficients in bold.

Table S3. Outputs (Beta and *p*-values) of hierarchical multivariate regression models on influencing factors of children deficit in viso-motoric development (%).

	β_1 (Social Index)	β_2 (Non-German %)	β_3 (Single Parent Household %)	β_4 (Kindergarten Attendance %)	β_5 (Natural Area %)	β_6 (Access Natural Area %)	β_7 (Per Capita Natural Area %)	R^2-Adjusted (%)
Model 1	**−0.46**	**−0.27**	0.18	**−0.36**				46.9
p values	0.009	0.093	0.128	0.043				
Model 2	**−0.42**	−0.18	**0.20**	**−0.35**	**−0.81**	0.11	**0.61**	62.5 *
p values	0.009	0.241	0.047	0.026	0.000	0.272	0.001	

Note: Significant coefficients in bold. * Change in R^2 is significant at 0.05.

Table S4. Outputs (Beta and *p*-values) of hierarchical multivariate regression models on influencing factors of children deficit in language development (%).

	β_1 (Social Index)	β_2 (Non-German %)	β_3 (Single Parent Household %)	β_4 (Kindergarten Attendance %)	β_5 (Natural Area %)	β_6 (Access Natural Area %)	β_7 (Per Capita Natural Area %)	R^2-Adjusted (%)
Model 1	**−0.435**	**0.309**	0.027	**−0.291**				89.0
p values	0.000	0.000	0.611	0.001				
Model 2	**−0.443**	**0.310**	0.030	**−0.280**	**−0.301**	0.027	**0.175**	91.7 *
p values	0.000	0.000	0.513	0.000	0.000	0.563	0.035	

Note: Significant coefficients in bold. * Change in R^2 is significant at 0.05.

References

1. EEA. Urban Atlas 2006. Available online: http://www.eea.europa.eu/data-and-maps/data/urban-atlas (accessed on 11 July 2012).

© 2016 by the authors; licensee MDPI, Basel, Switzerland. This article is an open access article distributed under the terms and conditions of the Creative Commons by Attribution (CC-BY) license (http://creativecommons.org/licenses/by/4.0/).
